# Percutaneous Peripheral Nerve Stimulation for Chronic Low Back Pain: Prospective Case Series With 1 Year of Sustained Relief Following Short‐Term Implant

**DOI:** 10.1111/papr.12856

**Published:** 2019-12-02

**Authors:** Christopher A. Gilmore, Leonardo Kapural, Meredith J. McGee, Joseph W. Boggs

**Affiliations:** ^1^ Center for Clinical Research Carolinas Pain Institute Winston Salem North Carolina U.S.A.; ^2^ SPR Therapeutics, Inc. Cleveland Ohio U.S.A.

**Keywords:** low back pain, percutaneous peripheral nerve stimulation, chronic pain, disability

## Abstract

**Introduction:**

Percutaneous peripheral nerve stimulation (PNS) provides an opportunity to relieve chronic low back pain and reduce opioid analgesic consumption as an alternative to radiofrequency ablation and permanently implanted neurostimulation systems. Traditionally, the use of neurostimulation earlier in the treatment continuum has been limited by its associated risk, invasiveness, and cost.

**Methods:**

Percutaneous PNS leads (SPRINT MicroLead) were placed bilaterally to target the medial branches of the dorsal rami nerves under image guidance. The percutaneous leads were connected to miniature wearable stimulators (SPRINT PNS System) for the 1‐month therapy period, after which the leads were removed. Pain and disability were assessed long‐term up to 12 months after lead removal.

**Results:**

Substantial, clinically significant reductions in average pain intensity (≥50% reduction as measured by the Brief Pain Inventory Short Form) were experienced by a majority of subjects (67%) at end of treatment compared to baseline (average 80% reduction among responders; *P* < 0.05, analysis of variance; *n* = 9). Twelve months after the end of PNS treatment, a majority of subjects who completed the long‐term follow‐up visits experienced sustained, clinically significant reductions in pain and/or disability (67%, *n* = 6; average 63% reduction in pain intensity and 32‐point reduction in disability among responders). No serious or unanticipated adverse events were reported.

**Conclusions:**

This study challenges the long‐held notion that a positive trial of PNS should be followed by a permanent implant in responders. Percutaneous PNS may serve as an effective neurostimulation therapy for patients with chronic low back pain and should be considered earlier in the treatment continuum as a motor‐sparing means of avoiding opioids, denervation, and permanently implanted neurostimulation systems.

## Introduction

Chronic low back pain (LBP) is a leading cause of disability among adults and is one of the most prevalent musculoskeletal conditions that is challenging to treat.^1^, ^2^, ^3^ Disability as a result of chronic LBP is a common complaint, as pain often decreases quality of life by interfering with function and reducing the patient’s ability to complete normal activities of daily living, such as walking, housework, or personal care.^4^, ^5^, ^6^ Further, because chronic LBP is difficult to diagnose and treat, it represents a significant healthcare burden. In many cases (up to 85%), chronic LBP may be nonspecific or have an unidentified cause.^7^ After LBP has become chronic (typically defined as pain lasting longer than 12 weeks), pain and disability can intensify as the result of a cycle of central sensitization, whereby changes in central pain processing result in hypersensitivity to normal inputs and persistent pain, even if injuries have healed.^8^, ^9^, ^10^, ^11^ It is the presence of both peripheral and central pain generators in chronic LBP that has made successful treatment with conventional methods challenging.^12^

### Chronic LBP and the Opioid Crisis

Despite changes in recent years to LBP treatment guidelines, which suggest that opioids should not be offered for the treatment of chronic LBP, opioids continue to be commonly prescribed and a diagnosis of chronic LBP is associated with an increased likelihood of opioid use and abuse.^13^, ^14^, ^15^ Because there is little evidence supporting the efficacy of opioids in treating chronic LBP, improving the patient’s ability to return to work, or reducing the need for other pain therapies,^16^ it is widely accepted that an effective, non‐opioid treatment is needed to treat chronic back pain and limit opioid use to prevent dependence and addiction.

### Treatments for Chronic LBP

Historically, the treatment paradigm for non‐opioid pain management has included a wide range of approaches of increasing invasiveness, from medications (eg, non‐opioid analgesics such as nonsteroidal anti‐inflammatory drugs, muscle relaxants, tricyclic antidepressants, or corticosteroids^17^, ^18^), to transcutaneous electrical nerve stimulation (TENS), physical therapy, and interventions such as anesthetic or steroid injections, radiofrequency ablation, permanently implanted neurostimulation or intrathecal drug delivery systems, or surgery.

Although transcutaneous electrical nerve stimulation (TENS; applied via surface electrodes) is used as a minimally invasive treatment option by patients, it may cause discomfort due to activation of cutaneous nerve endings or skin irritation at the stimulation intensities required to activate the deep pain‐relieving nerve fibers, leading to low rates of patient compliance or ineffective treatment at comfortable intensities.^19^, ^20^, ^21^, ^22^, ^23^, ^24^, ^25^, ^26^, ^27^, ^28^, ^29^, ^30^, ^31^, ^32^, ^33^, ^34^ Interestingly, although some payer coverage policies require that a patient have a positive response to TENS prior to undergoing peripheral nerve stimulation (PNS), TENS has not been found to be a reliable predictor of PNS efficacy.^35^ Physical therapy can reduce back pain and disability, and thereby opioid usage, but patients may be unwilling or unable to comply with prescribed regimens.^36^, ^37^, ^38^, ^39^, ^40^, ^41^, ^42^ Anesthetic and/or corticosteroid injections usually only provide short‐term relief and must be repeated.^43^, ^44^, ^45^, ^46^, ^47^, ^48^ Radiofrequency ablation can provide pain relief in well‐selected patients with facetogenic pain, but is highly dependent on physician expertise, destroys nerve tissue and/or denervates key paraspinal muscles, and is often followed by a return of pain after several months.^49^

Surgery and many permanently implanted neurostimulation systems can be highly invasive, complex, irreversible, and carry risks of complications. Surgical procedures (eg, spinal fusion, disc replacement) for back pain that attempt to repair physical deformities frequently fail to reduce pain or disability^50^, ^51^, ^52^ and may result in failed back surgery syndrome^53^ or a need for reoperation.^54^, ^55^, ^56^, ^57^ Permanently implanted neurostimulation (eg*,* spinal cord stimulation [SCS]) systems can reduce pain, opioid use, and disability,^58^, ^59^, ^60^, ^61^, ^62^, ^63^, ^64^, ^65^, ^66^, ^67^ but traditionally multifactorial etiology pain, as is common among those with chronic LBP, has been difficult to successfully treat conventionally.^68^, ^69^, ^70^ Due in part to the invasiveness, risks of the surgery and implantation of leads near the spinal cord, high complication rate,^71^, ^72^, ^73^, ^74^, ^75^, ^76^ and associated expense, SCS is typically relegated to a treatment of last resort (ie*,* only employed after other therapies have failed) and only used in about 5% of candidates.^77^

**Figure 1:**
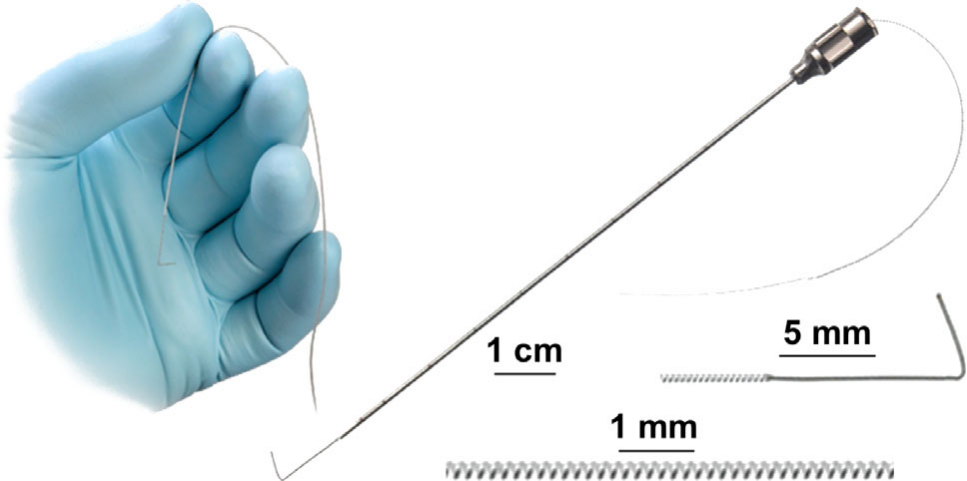
Percutaneous peripheral nerve stimulation (PNS) lead used for treatment of chronic low back pain (LBP). Percutaneous PNS was delivered using a coiled, fine‐wire MicroLead (SPR Therapeutics, Inc.) for 1 month for the treatment of chronic LBP. Figure used with permission from SPR Therapeutics, Inc.

An effective and less invasive system is needed that does not have the costs, risks, complications, and delayed care associated with previous therapies,^78^, ^79^ especially if such a system may permit short‐term use to interrupt the cycle of chronic pain and provides long‐term pain relief that prevents the recurrence of pain or the need for surgery or a permanent implant.^80^, ^81^ Percutaneous PNS consists of 1 or 2 fine‐wire leads (Figure 1), which are implanted via a percutaneous introducer and connected to a miniature wearable stimulator that is programmed by the clinician and adjusted by the patient. The system enables delivery of electrical stimulation to nerves innervating the region of pain (eg*,* the low back), while avoiding the challenges associated with permanently implanted neurostimulation systems. Studies suggest that use of neurostimulation earlier in the treatment continuum could improve patient outcomes, for example, by reducing the number of hospitalizations and clinic visits, or reducing opioid usage.^82^ An effective option is needed earlier in the treatment continuum that can reduce pain, opioid use, and disability, with the benefits of a neurostimulation system, while avoiding the need for a permanently implanted system and the associated risks of complications.^78^, ^79^ Percutaneous PNS provides an opportunity as an earlier neurostimulation intervention that may preclude the need for opioids, denervation, surgery, and permanently implanted systems, particularly given evidence of long‐term relief in patients with chronic pain.^80^, ^81^, ^83^, ^84^, ^85^, ^86^, ^87^, ^88^, ^89^, ^90^, ^91^, ^92^, ^93^, ^94^, ^95^, ^96^, ^97^ A recently published double‐blinded randomized controlled trial (RCT) assessing the efficacy of percutaneous PNS in the treatment of chronic postamputation pain demonstrated significant and durable pain relief and improvement in quality of life with results sustained through 1 year.^81^, ^97^ The present investigation was designed as a prospective case series study to determine the feasibility of generating similar sustainable reductions in pain and disability and improvements in quality of life in patients with LBP using the same device.

## Methods

Individuals with chronic LBP were screened for enrollment in a prospective case series study approved by U.S. Food and Drug Administration (FDA) investigational device exemption and the institutional review board (IRB; Quorum IRB, Seattle, WA, U.S.A.; registered on ClinicalTrials.gov), and written informed consent was obtained from participants. Although the device was investigational at the time of enrollment, the SPRINT PNS System (SPR Therapeutics, Inc., Cleveland, OH, U.S.A.) has since received FDA 510(k) clearance and is indicated for use “up to 60 days in the back and/or extremities for: (1) Symptomatic relief of chronic, intractable pain, post‐surgical and post‐traumatic acute pain; (2) Symptomatic relief of post‐traumatic pain; and (3) Symptomatic relief of post‐operative pain. It is not intended to treat pain in the craniofacial region.”

Participants must have had chronic axial LBP (ie, lumbar pain with a duration ≥ 12 weeks) with at least 4 weeks of stable analgesic medication usage. Subjects completed a 7‐day baseline diary by recording their daily average and worst pain intensities on a numeric rating scale from 0 to 10 (Brief Pain Inventory Short Form, BPI‐3, BPI‐5). To enroll, subjects must have had a baseline average BPI‐5 score (average pain intensity) ≥ 4. Subjects were excluded from participation if they had any of the following: radicular pain; previous lumbar surgery; signs of a serious underlying cause of LBP (eg, cancer, chronic infection, metabolic bone disorder); anesthetic injections within 3 months of baseline; radiofrequency ablation within 6 months of baseline; conditions such as fibromyalgia, multiple sclerosis, or spinal cord injury; pending litigation; signs of infection on or around the low back or other conditions that increase the risk for infection; allergy to medical‐grade adhesives or tapes; an implanted pacemaker/defibrillator or neurostimulator; body mass index (BMI) ≥ 40; or depression (score > 20 on the Beck Depression Inventory [BDI‐II]). Due to the feasibility nature of this prospective case series study, participants were not required to have previously received a specific LBP diagnosis or to have completed prior diagnostic testing or imaging confirming a particular etiology of LBP.

Percutaneous, open‐coil PNS leads (MicroLead; SPR Therapeutics) were implanted bilaterally under sterile conditions with the subject in a prone position to target the medial branches of the dorsal rami nerves. The location for lead placement in each participant was selected following physical examination and manual palpation by the physician to determine the location of axial LBP. Leads were implanted at the segmental level corresponding with the center of each subject’s painful region, confirmed by ultrasound.

**Figure 2:**
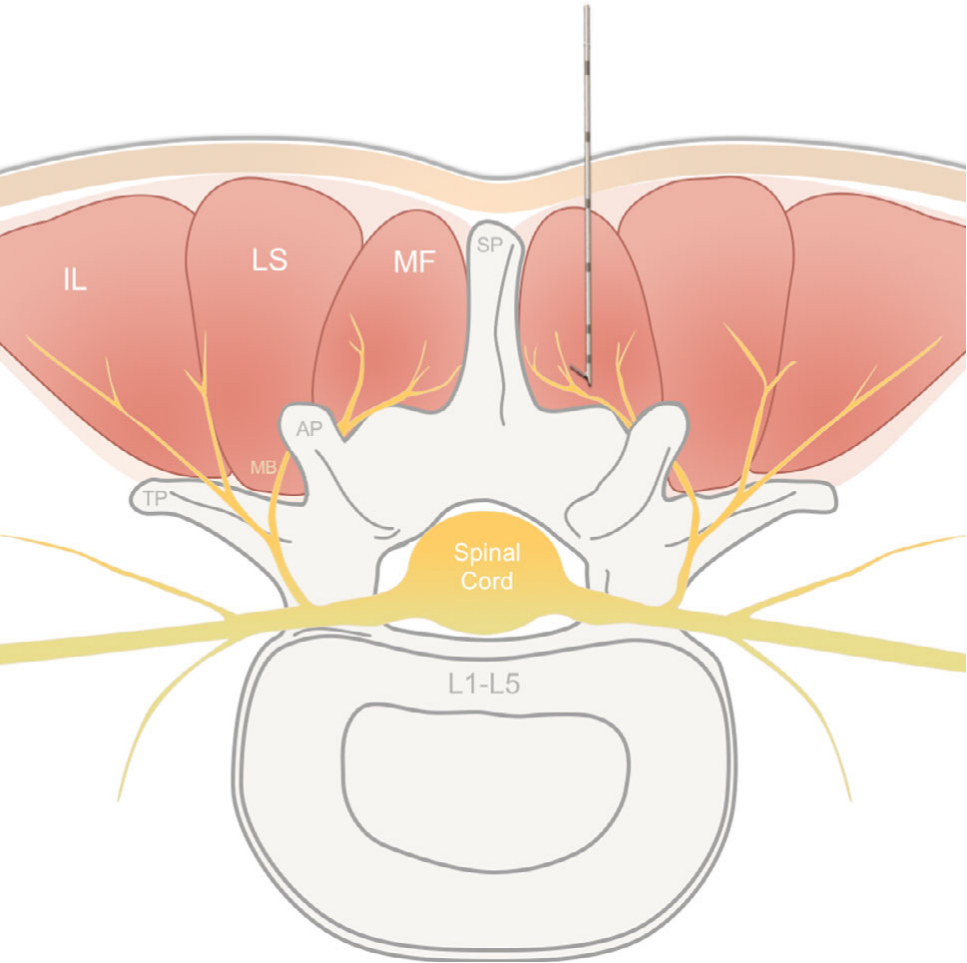
Anatomical target of percutaneous peripheral nerve stimulation lead placement for treatment of chronic low back pain. Percutaneous fine‐wire leads (SPRINT MicroLead, SPR Therapeutics, Inc.) were placed to target the medial branch (MB) of the dorsal ramus, medial and inferior to the facet joint in the center of the region of pain. Selective activation of the multifidus (MF) overlapping the area of pain confirmed appropriate lead placement. A cross‐sectional view of the lumbar paraspinal anatomy is shown, with the lead and introducer placed targeting the MB of the dorsal ramus. AP, articular process; IL, iliocostalis; LS, longissimus; SP, spinous process; TP, transverse process.

To identify the correct location of lead placement, a stimulating probe was first placed into the tissue under ultrasound guidance, using known anatomical landmarks to target the medial branches of the dorsal rami after the nerve exits the intervertebral foramen as it lies along the lamina, medial and inferior to the facet joint (Figure 2). Stimulation of the nerve target (medial branch of the dorsal ramus nerve) was confirmed with selective activation of the lumbar multifidus and comfortable contractions overlapping the region of pain. Upon successful stimulation of the medial branches of the dorsal ramus, the stimulating probe was removed and the percutaneous PNS lead was implanted with the same approach via a preloaded introducer. Multifidus contractions at the final location of PNS lead placement were verified with ultrasound visualization and patient‐reported sensations of stimulation.

The percutaneous leads remained implanted for the duration of the 1‐month therapy and were connected to the miniature wearable stimulators (SPRINT PNS System; SPR Therapeutics). Stimulation was programmed to selectively stimulate the medial branches of the dorsal rami, the nerves innervating the multifidi, to result in comfortable cycling activation in the region of pain (frequency = 12 Hz). The stimulators were programmed such that each subject received a customized range of intensities, which generated strong, but comfortable sensations. Subjects were encouraged to use stimulation for 6 hours per day for each day of the 1‐month treatment period, while continuing their normal daily routines and activities.

During treatment with percutaneous PNS, subjects were not allowed to engage in or receive any other treatments for LBP, apart from their baseline medications. Each week of treatment, subjects recorded daily pain levels and analgesic medication consumption in diaries and returned to the clinic for evaluation and assessments (eg*,* disability via the Oswestry Disability Index [ODI]; patient global impression of change [PGIC]; adverse events). Leads were removed at the end of the 1‐month therapy (end of treatment). Subjects later returned to the clinic for follow‐up visits and assessments at 3, 6, and 12 months after the end of treatment.

## Results

This report reviews the long‐term results of 9 subjects enrolled in this prospective case series study who met the eligibility criteria and received percutaneous PNS for the treatment of chronic LBP. This report is the first to describe the sustained results among responders at 1 year after the end of the PNS therapy.

### Baseline Characteristics and PNS Treatment

At enrollment, subjects were on average 53.3 years old with an average BMI of 28.9. The average duration of chronic LBP prior to enrollment was 10 years, despite use of several therapies for LBP, such as opioids, non‐opioids, physical therapy, and injections. After the physical examination and review of LBP‐related history at the baseline visit, the etiology of chronic LBP for 1 participant was determined to be degenerative disc disease, but a majority of the participants (*n* = 8/9) had nonspecific axial LBP, or pain of an unknown cause. Each subject underwent implantation of fine‐wire percutaneous PNS leads without complications, as outlined in the Methods section. All subjects received bilateral percutaneous PNS with 2 leads, except for 1 subject with unilateral, right‐sided pain who received only 1 PNS lead ipsilateral to the side of pain. Subjects reported that stimulation of the medial branches of the dorsal rami nerves resulted in comfortable sensations in regions overlapping their LBP.

Correct lead positioning was confirmed during weekly visits by inspection of the lead exit site, queries for changes in sensation with stimulation, and evaluation of stimulation thresholds for muscle activation. At the end of the 1‐month therapy, the percutaneous PNS leads were removed without discomfort or complication.

### Results at End of PNS Treatment

After 1 month of treatment with percutaneous PNS, substantial, clinically significant reductions in average pain intensity (≥ 50% as measured on the BPI‐5) compared to baseline were experienced by a majority of subjects (67%, average 80% reduction among responders; *P* < 0.05, analysis of variance; *n* = 9; 95% confidence interval [CI] [0.36, 0.97]). Clinically significant reductions in disability (≥ 10‐point reduction on the ODI) were also experienced by a majority of subjects (67%, average 22.9‐point reduction among responders; *n* = 9; 95% CI [0.36, 0.97]).^6^ These subjects also reported substantial reductions in analgesic usage, as 83% of subjects (*n* = 5/6 taking medications at baseline; 95% CI [0.53, 1.13]) reported a 50% or greater reduction in total analgesic medication usage (ie, both opioids and non‐opioids). Importantly, all subjects also either successfully avoided opioids during treatment with PNS (*n* = 8) or ceased using opioids with PNS (*n* = 1). On average, subjects reported that their quality of life was “much improved” with PNS treatment (PGIC, on a scale of very much worse to very much improved). A subject satisfaction survey assessed at the end of treatment found that a majority (88%) would recommend PNS to a friend with LBP and a majority (88%) also preferred to use PNS over analgesic medications (*n* = 8; 95% CI [0.65, 1.10]).

### Sustained Results at 1 Year Post‐Treatment

**Figure 3:**
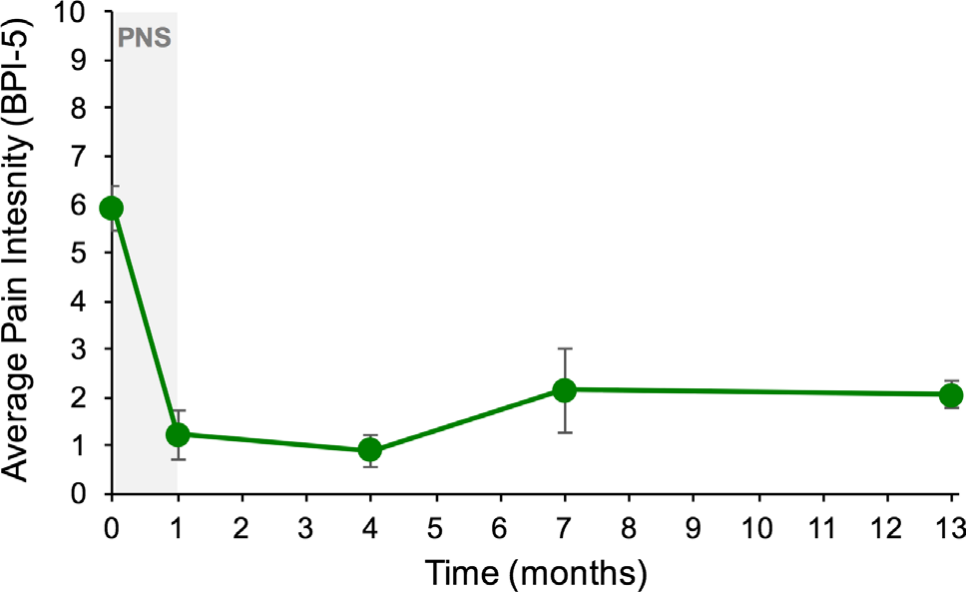
Average pain intensity among responders over time. Subjects experiencing a clinically significant reduction in pain at the end of treatment (≥ 50% reduction) continued to experience sustained results long term, through at least 12 months after the end of peripheral nerve stimulation (PNS) treatment. The average reduction among responders 1 year after end of treatment with PNS was 63%. BPI‐5, Brief Pain Inventory Short Form.

**Figure 4:**
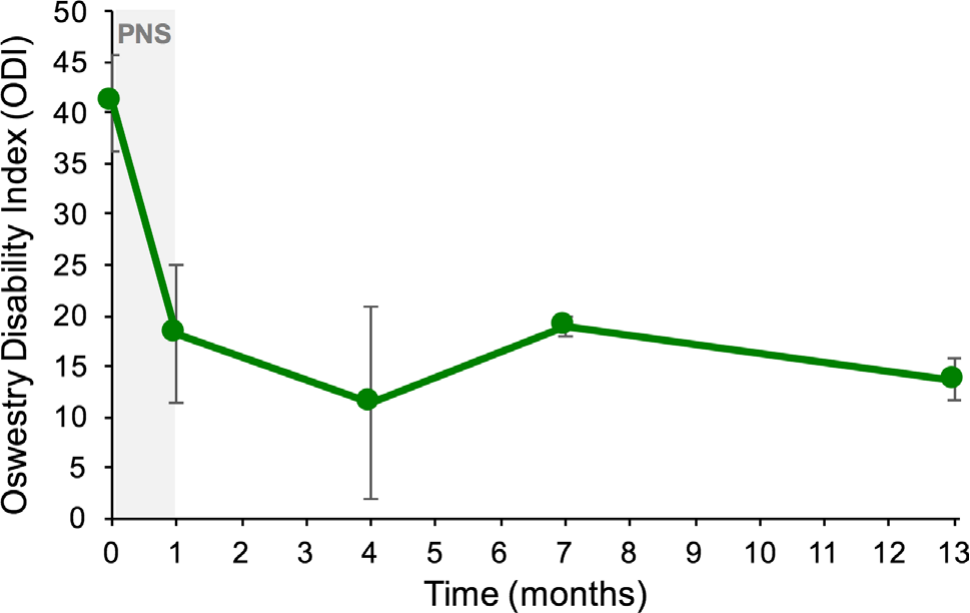
Oswestry Disability Index results among responders over time. Subjects experiencing a clinically significant reduction in disability (≥ 10 points) continued to experience sustained results long term, through at least 12 months after the end of peripheral nerve stimulation (PNS) treatment. The average reduction in disability among responders 1 year after the end of treatment with PNS was 32 points. A 10‐point improvement in the Oswestry Disability Index score is considered clinically significant.

Twelve months after the end of PNS treatment, a majority (67%) of subjects completing the long‐term follow‐up visits reported clinically significant reductions in pain intensity and/or disability (*n* = 6; 95% CI [0.28, 1.04]). Among those completing long‐term follow‐up visits, 50% experienced substantial clinically significant reductions (≥ 50%) in pain intensity, which were sustained at 12 months (*n* = 6; average 63% reduction in pain intensity among responders; Figure 3). Further, 50% also experienced clinically significant reductions in disability at 12 months after the end of PNS treatment (average 32‐point reduction in ODI score among responders; Figure 4).

A year after treatment with PNS, a majority of subjects (83%) reported improvement in their quality of life since enrollment due to PNS (PGIC; 95% CI [0.54, 1.13]). Results of a subject satisfaction survey at 12 months after end of treatment revealed that all subjects completing the 12‐month follow‐up visit (100%) were either satisfied or very satisfied with the pain relief they received following stimulation therapy. All subjects (100%) also reported that had this method of percutaneous PNS been previously available, they would prefer to have pursued PNS earlier in the treatment for their LBP.

### Safety

No serious or unanticipated adverse events occurred in this study of percutaneous PNS for the treatment of chronic LBP. Reports of skin irritation were the only adverse events related to the device or procedure and occurred in 2 subjects. These subjects reported redness and itching at the location where dressings were located on the skin. These subjects were provided with latex‐free dressings to help reduce the risk for skin irritation.

## Discussion

This report is the first to explore the long‐term effects of percutaneous PNS of the medial branches of the dorsal rami for the treatment of chronic LBP 1 year after the end of treatment and lead removal. The medial branches of the dorsal ramus were selected as the target of percutaneous PNS with this approach due to their purported role in chronic nonspecific LBP and innervation of the multifidus overlapping regions of axial LBP.^98^ While the use of ultrasound for needle guidance for other pain management approaches, such as anesthetic nerve blocks,^99^ has become common, this study demonstrates the feasibility of successfully targeting the medial branches of the dorsal rami with percutaneous PNS under ultrasound guidance, without requiring fluoroscopy, as is commonly utilized in other neurostimulation applications.

Percutaneous PNS treatment produced sustained, clinically meaningful improvements in chronic LBP and secondary outcomes at 12 months. A majority of subjects experienced substantial (≥50%) reductions in average pain intensity with treatment, with an average reduction of 80% among responders. A majority of subjects also experienced significant reductions in LBP‐related disability (≥10‐point reduction in ODI score), with an average 23‐point reduction among responders with treatment. Importantly, these clinically significant reductions were sustained long‐term at 1 year after PNS lead removal. Of subjects completing long‐term follow‐up visits at 12 months after the end of treatment, a majority (67%) reported sustained substantial reductions in pain intensity and/or disability.

Percutaneous PNS enabled reductions in both opioid and non‐opioid analgesic medication consumption during treatment, which were sustained in the long‐term follow‐up. Given the ongoing opioid crisis, it was important to note that all subjects also successfully either avoided opioid consumption (*n* = 8) or ceased opioid consumption (*n* = 1) with PNS treatment, which was sustained long term. Subjects’ improvements in chronic LBP were also corroborated by improvements in quality of life via the PGIC, demonstrating the potential for PNS to significantly improve quality of life and physical functioning, by relieving pain long‐term, out to at least 1 year after treatment.

### Proposed Mechanism of Action

The sustained improvements reported here are consistent with results from previously published studies where percutaneous PNS provided sustained clinically significant pain relief in several other pain conditions, such as chronic shoulder pain, neuropathic pain, post‐surgical pain, and back pain.^8^, ^9^, ^10^, ^11^, ^12^ One of the key elements of the mechanism of action proposed to be responsible for the sustained analgesic effects of percutaneous PNS is thought to be the modulation of central sensitization, often thought to occur among patients with chronic back pain and other painful conditions with both nociceptive and neuropathic characteristics.^8^, ^9^, ^100^ In addition to stimulation of afferent fibers, which engage the gate mechanism directly to reduce pain signaling, stimulation of efferent nerve fibers activates muscles and thereby generates physiological proprioceptive afferent signals from the muscle spindles and Golgi tendon organs activated in those muscles.^101^, ^102^ Together, these afferent signals may help to normalize or partially reverse membrane excitability of neurons and circuits in the pain processing pathways.^103^ This reduction in pain signals with PNS may also disrupt the cycle of centrally mediated pain, permitting greater levels of activity, which may further reduce pain via activity‐dependent neuroplasticity even long after therapy delivery has ended.^104^, ^105^, ^106^ This mechanism could explain the pain reduction experienced during treatment with percutaneous PNS, and it may also support the durability of the effect on pain and improvements in disability long term.^14^

Percutaneous PNS may serve as an effective early intervention neurostimulation therapy for the treatment of LBP that preserves motor function and obviates the need for a permanent implant. The fine‐wire percutaneous PNS leads were designed to reduce invasiveness, adverse events, and some of the technical challenges that were previously associated with traditional systems and applications for PNS. Because this PNS system was designed purposely for use as a percutaneous therapy, it has an excellent safety profile and fewer complications than permanently implanted neurostimulation systems.^15^, ^16^, ^17^, ^18^ Studies suggest that use of neurostimulation earlier in the treatment continuum could also improve patient outcomes, for example, by reducing the number of hospitalizations and clinic visits, or reducing opioid usage.^82^ This approach of percutaneous PNS provides a unique opportunity to be used as an earlier neurostimulation intervention that may preclude the need for opioids, denervation, and permanently implanted neurostimulation systems, due to the less invasive nature of the intervention in conjunction with evidence of long‐term relief in patients with many types of chronic pain.^80^, ^81^, ^83^, ^84^, ^85^, ^86^, ^87^, ^88^, ^89^, ^90^, ^91^, ^92^, ^93^, ^94^, ^95^, ^96^, ^97^ In addition to 2 previously published RCTs using this same device for the treatment of chronic shoulder pain,^86^, ^94^ a third double‐blinded RCT was recently published and demonstrated successful relief of chronic neuropathic pain and improvement in quality of life, with results sustained through 1 year.^97^

### Limitations

Although the results presented here are promising and consistent with previous studies of percutaneous PNS for other types of pain,^80^, ^81^, ^83^, ^84^, ^85^, ^86^, ^87^, ^88^, ^89^, ^90^, ^91^, ^92^, ^93^, ^94^, ^95^, ^96^, ^97^ this study has limitations, which should be considered in interpretation of the results. In particular, the population size was limited (*n* = 9) and did not include a control group or explore placebo effect; additional studies could help confirm these results in a larger population of patients, including studies that might compare the effects of percutaneous PNS to other standard interventional approaches used for patients with chronic LBP. Because chronic LBP can include a heterogeneous population (eg, facetogenic, discogenic, arthritic, or myofascial pain) and the selection criteria for inclusion in this study were broad, additional studies and analyses of larger populations, including larger, prospective multicenter case series studies, may determine LBP subtypes or characteristics that are more likely to benefit from percutaneous PNS, as well as if specific types of diagnostic tests or imaging are predictive of success.

The results from the present investigation suggest that percutaneous PNS can produce significant reductions in pain and disability and improvements in quality of life among patients with chronic LBP out to at least 1 year after treatment. The large population of patients with chronic LBP is in need of less invasive, non‐opioid pain management therapies that could be provided earlier in the treatment continuum, before more invasive, destructive, or expensive therapies, such as surgery or permanent neurostimulation system implantation. As such, percutaneous PNS may offer substantial advantages and an opportunity to shift the paradigm by offering effective neurostimulation to these patients earlier in the treatment continuum.

## Conclusion

The results reported here showing sustained relief of chronic LBP and disability for at least 12 months highlight the potential for percutaneous PNS to obviate the need for more invasive permanently implanted systems. This is consistent with previously published studies and RCTs of percutaneous PNS in other pain indications (eg*,* neuropathic pain, chronic shoulder pain), where clinically significant reductions in pain and improvements in pain‐related disability were also sustained long‐term.^80^, ^81^, ^83^, ^84^, ^85^, ^86^, ^87^, ^88^, ^89^, ^90^, ^91^, ^92^, ^93^, ^94^, ^95^, ^96^, ^97^ Together, these studies reveal the potential for percutaneous PNS to be used as an alternative to existing treatment modalities for chronic pain, effectively reducing pain and opioid use, while reducing disability and invasiveness. This approach has the potential to significantly influence the care continuum for chronic back pain by providing the benefits of an effective neurostimulation therapy to patients earlier than has been previously possible.

## Conflicts of Interest

C.G. and L.K. have consulted for SPR Therapeutics. M.M. and J.B. are employees of SPR Therapeutics.
